# Tear levels of SFRP1 are significantly reduced in keratoconus patients

**Published:** 2013-02-25

**Authors:** Jingjing You, Chris Hodge, Li Wen, John W. McAvoy, Michele C. Madigan, Gerard Sutton

**Affiliations:** 1 Save Sight Institute, Discipline of Clinical Ophthalmology, The University of Sydney, Australia; 2 Vision Eye Institute, Australia; 3 School of Optometry & Vision Sciences, UNSW, Australia; 4 Auckland University, New Zealand

## Abstract

**Purpose:**

To measure secreted frizzled-related protein 1 (SFRP1) levels in human tears and to investigate tear SFRP1 as a potential biomarker for keratoconus (KC).

**Methods:**

Tears were collected from control (n=33) and KC patients (n=33) using micropipette tubes. Total tear protein was measured using a FluoroProfile Protein Quantification kit. An in-house enzyme-linked immunosorbent assay (ELISA) was developed to measure SFRP1 in control and KC tears. Statistical analyses of age, gender, the association of SFRP1, and total tear protein with KC were conducted.

**Results:**

Tear SFRP1 was significantly decreased in KC, compared to age-matched controls (3.41 ng/μl±3.12 versus 5.55 ng/μl±5.62, respectively; p=0.039). Conversely, total tear protein was significantly increased in KC, compared to age-matched controls (12.38 μg/μl±4.76 versus 9.40 μg/μl±3.88, respectively; p=0.038). The ratio of SFRP1/total tear protein was also found to be significantly decreased in the KC group (p=0.007). No significant association between tear SFRP1 and total tear protein was detected.

**Conclusions:**

Tear SFRP1 was significantly decreased in age-matched KC versus control patients, and may be further reduced in moderate KC. Tear-SFRP1 levels alone do not provide an obvious biomarker for KC; however, our results provide further evidence that tear-protein profiles are altered in KC, and suggest the involvement of SFRPs in the pathogenesis of KC.

## Introduction

Keratoconus (KC) is the most common primary degenerative corneal disease, with a prevalence of around 1 in 2,000 worldwide [1]. The condition often presents bilaterally with asymmetric progression, leading to corneal thinning and the development of an irregular corneal shape. Although it does not cause blindness, KC has been shown to significantly reduce perceived quality of life [2].

The clinical symptoms of KC vary depending on the stage of progression. In the early stages, clinical findings may be limited to specialized diagnostic tests such as corneal topography. In more advanced cases, visual acuity may not be adequately corrected with optical aids. Various management strategies and treatments are available, including soft and rigid gas-permeable contact lenses for mild to moderate cases and surgical interventions such as collagen cross-linking, intracorneal ring segments, and corneal transplantation for moderate to severe cases [3]. The major challenge for clinicians is to determine which treatment is most appropriate for the individual patient. While clinical and surgical experience is integral to patient management, it is limited by our understanding of the etiology and pathogenesis of KC. Biomarkers have been widely used in other diseases, such as cancer and diabetes, and a reliable biomarker for detecting patients before clinical symptoms associated with KC are reported would be clinically useful in providing more effective prognostication and options for treatment and management.

We previously detected significantly increased mRNA and protein expression of secreted frizzled-related protein 1 (SFRP1) in KC corneal epithelium, compared to controls [4]. The SFRP family of glycoproteins inhibits Wnt signaling pathways by binding to Wnts or Frizzled (Fzd) proteins, preventing formation of the Wnt-Fzd complex, essential for the activation of Wnt pathways [5]. SFRPs may also function independently of Wnt signaling pathways [6]. Altered SFRP1 expression has been reported to be associated with cell apoptosis in various conditions, including cancer [7], periodontitis [8], and bone disease [9]. In KC, apoptosis of the anterior stromal keratocytes is associated with loss of stromal extracellular matrix and corneal thinning [10].

Tears are increasingly used as a source for discovering protein biomarkers for both ocular and systemic diseases. We previously developed an immunoblotting technique to relatively quantify proteins in biological fluids such as tears [11]. To validate this technique, we analyzed a small number of KC and control samples and found relatively less tear SFRP1 in KC, compared to controls [11]. This contrasted with our earlier findings in KC corneal epithelium [4], so a further quantitative analysis of tear SFRP1 was undertaken. In the current study, we developed an enzyme-linked immunosorbent assay (ELISA) to measure the absolute SFRP1 concentration in tears. Using a sample size appropriate to establishing statistical significance and taking into consideration age and gender, we aimed to examine the SFRP1 levels and total protein concentration in KC and control tears to investigate the potential of SFRP1 as a biomarker for KC.

## Methods

### Samples

Tears from control (n=33) and KC patients (n=33; Table 1) were collected using a 10 µl Blaubrand intraMARK micropipette (Brand GMBH, Wertheim, Germany) placed gently onto the corner of the eye, avoiding contact with the conjunctival surface. Total tear collection time was <15 min. The minimum total sample size (including both cohorts) required for a power of 80% was n=64.

**Table 1:** No significant differences were detected using Chi-Square analysis between gender for control and KC groups, and within KC grades.

Comparison between KC and Control Groups			
Group	Gender		Chi-Square
	Female	Male	
Control (n=33)	18	15	p=0.971
KC (n=33)	14	19	

Comparison within KC grades			
KC Grade*	Gender		Chi-Sqaure
	Female	Male	
1	0	1	p=0.950
2	4	6	
3	4	4	
4	6	8	
Total	14	19	

*KC Grade Grade 1: subclinical form diagnosed by corneal topography; ~6/6VA (Visual Acuity) with spectacle correction. Grade 2: early form; mild corneal thinning; corneal scarring absent. Grade 3: moderate form; corneal scarring and opacities absent; Vogt’s striae; Fleischer’s ring; <6/6 VA with spectacle correction, but ~6/6VA with contact lens correction; irregular astigmatism 2.00 to 8.00D; significant corneal thinning. Grade 4: severe form; <6/7.5 VA with contact lens correction; severe corneal thinning and Munson’s sign.

All KC patients were recruited from the Vision Eye Institute (NSW, Australia), and were previously diagnosed based on clinical signs and corneal topography. KC patients were further classified as Grade 1 (mild) to Grade 4 (severe; Table 1). Control subjects were recruited from staff and volunteer students in the Save Sight Institute (NSW, Australia) and Vision Eye Institute. Only participants with no known eye diseases, no history of eye surgery or trauma, and no KC were included as controls in the study. The gender and age of each sample was recorded (Table 1 and Table 2). The study was approved by the Sydney Eye Hospital Human Research Ethics Committee, and informed consent was obtained before tear collection. All procedures were in accordance with the Declaration of Helsinki.

**Table 2:** Age Distribution for Control and KC Patients. A significant difference was detected between control and KC groups, no statistical analysis was performed within KC groups due to the small sample size.

Groups	Age (years)			Mean±SD^a^
	<20	20 to 30	>30	
Control	0	20 (60%)^b^	13	30.9±7.5
KC	7	19 (58%)^b^	7	25.8±6.7

Age distribution within KC grade				
KC Grades	Age (years)			Mean±SD^c^
	<20	20 to 30	>30	
1	0	0	1	38
2	4	4	2	24.6±8.2
3	2	5	1	24±5.6
4	1	10	3	26.7±5.5

^a^Kruskal–Wallis test, p=0.003. ^b^Percentage of total control and KC samples between 20 to 30 years of age. ^c^For KC Grade 1, n=1 and statistical analysis was not performed. We can only note that there was no obvious difference in age for KC Grade 2 to 4. SD: standard deviation.

### Secreted frizzled-related protein 1 ELISA assay

Tear SFRP1 was measured using ELISA. A 96-well MaxiSorp flat-bottom plate (NUNC, Roskilde, Denmark) was coated with 100 µl of goat polyclonal antihuman SFRP1 antibody (Abnova, Taipei, Taiwan) diluted in carbonate buffer (0.15 M sodium carbonate [BDH Merck, Poole Dorset, UK]/0.35 M sodium bicarbonate [BDH], pH 9.6) to a final concentration of 1 ng/µl. The plate was sealed and incubated overnight at 4 °C, with shaking. After incubation, the plate was equilibrated to room temperature, with shaking for 1 h, washed four times phosphate-buffered saline (PBS, AMRESCO, Solon, OH) with 0.05% Tween-20 ( Research Organics INC, Cleveland, OH), and tapped dry. After washing, 300 µl of blocking buffer (3% bovine serum albumin [BSA] in PBS) was added to each well and incubated at room temperature, with shaking for 2 h. The plate was washed as previously described; next, 100 µl of diluted standards and tear samples were added to each coated well and incubated at 4 °C overnight, with shaking. Serial diluted recombinant SFRP1 (Abcam, Cambridge, UK; 0, 0.002, 0.005, 0.01, 0.016, 0.05, and 0.1 ng/µl) in PBS was used to optimize a linear standard curve for the ELISA. Tear samples were diluted 1:200, 1:300, or 1:400 in PBS to ensure that the final optical density was within the linear range of the standard curve. After incubation, the plate was equilibrated to room temperature and washed as above. Diluted in PBS to a final concentration of 2 ng/µl, 100 µl of rabbit polyclonal antihuman SFRP1 (Abcam) were added to each well and incubated at 37 °C for 30 min, and at room temperature for 1 h. The plate was then washed and incubated with 100 µl of HRP-conjugated goat antirabbit IgG (Millipore, Billerica, MA) that was diluted 1:15,000 in PBST at 37 °C in the dark for 1 h. The plate was then washed and tapped dry. Added to each well were 100 µl of 1-Step Slow TMB-ELISA (Thermo Scientific, Billerica, MA), followed by incubation in the dark for 20 min; finally, the reaction was stopped with 100 µl of 2 M sulphuric acid. Optical density was measured at 450 nm (reference wavelength 570 nm) using a Tecan Safire^2^ microplate reader and Magellan software (Tecan, Männedorf, Switzerland). All samples were tested in triplicate, and two additional samples were included in each ELISA as quality controls.

### Total tear-protein assay

Total protein concentration was quantified using a FluoroProfile Protein Quantification kit (Sigma-Aldrich, St. Louis, MO) as described previously [11]. Briefly, serial dilutions of BSA were prepared to construct the standard curve. Tears were diluted 1 in 400 with MilliQ water, and 50 μl of each sample and standards were added to a 96-well flat-bottom PS-microplate (NUNC) in triplicate. An equal amount of FluoroProfile fluorescent reagent was added to each well, and the plate incubated at room temperature in the dark for 30 min. Fluorescent intensities were measured with excitation wavelength 510 nm and emission wavelength 620 nm, using a Tecan Safire^2^ microplate reader and Magellan software (Tecan). Tear-protein concentrations were calculated from the standard curve.

### Statistical analysis

Normality of age, total tear-protein and tear-SFRP1 levels were examined using the Shapiro-Wilk test. The Student *t* test was used for normally distributed data, and the Kruskal-Wallis test was used for data not normally distributed. The chi-square test was used to examine associations between gender and groups, and within KC grades. Total tear protein and tear SFRP1 were compared between control and KC groups. A p value of <0.05 was considered statistically significant. All statistical analyses were performed using IBM SPSS Statistics 20 software (IBM, Armonk, NY).

## Results

### Gender and age associations

Chi-square tests showed no significant differences in gender between the control and KC groups, or between KC grades (Table 1). Although the average age of controls was significantly greater than KC patients (p=0.003), the majority of tear samples were collected from patients between 20 and 30 years of age (Table 2).

### SFRP1 and total protein levels

**Figure 1:**
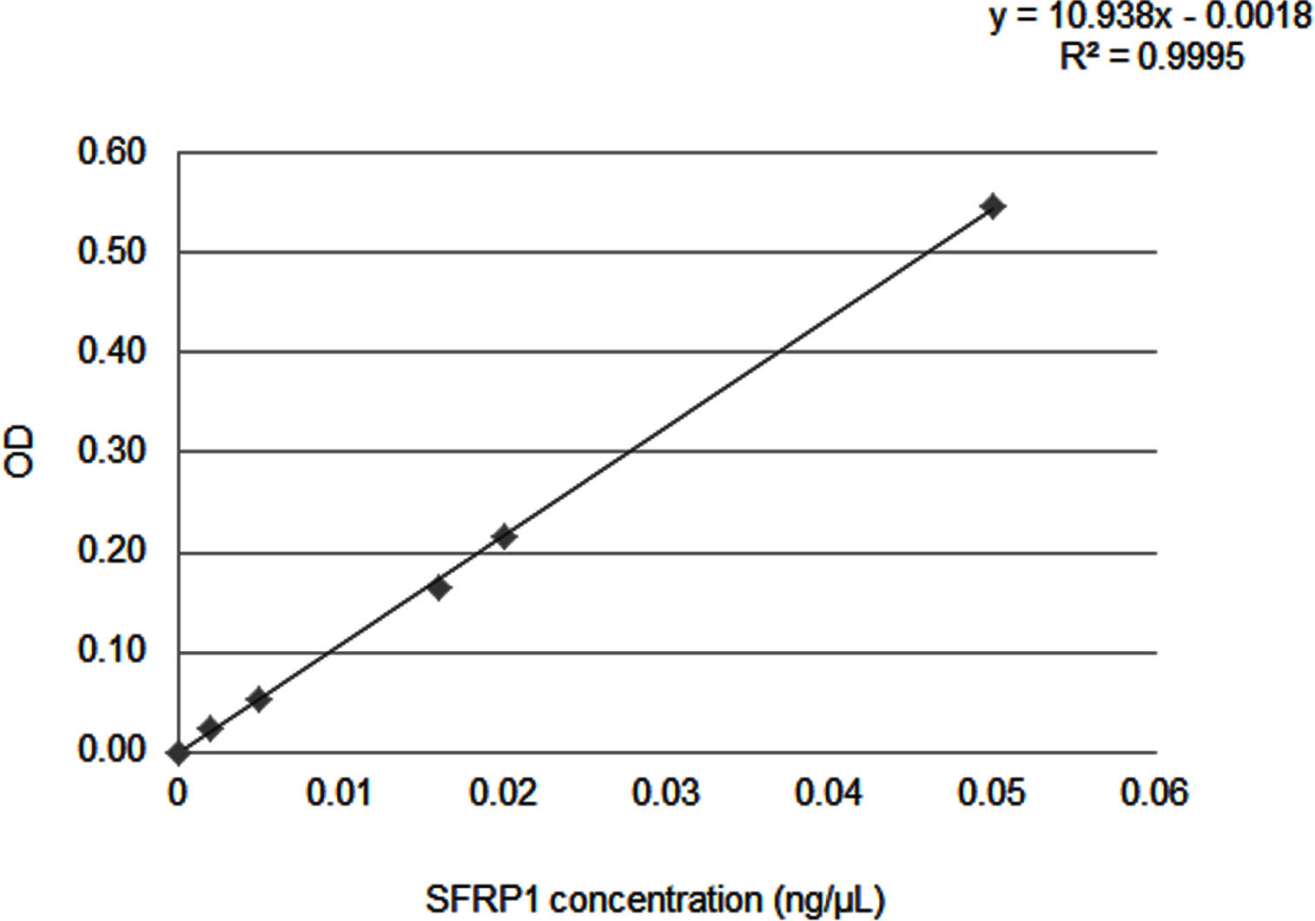
A representative standard curve for the tear secreted frizzled-related protein 1 (SFRP1) ELISA, with a correlation coefficient - R^2^-close to 1 (R^2^=0.9995). A high R^2^ shows that the standard curve is reliable and can be used for sequential calculations.

We detected SFRP1 to 2 pg/µl using the in-house ELISA (Figure 1). The ELISA intra- and inter-assay coefficient of variation (CV) percentages were <10% and <15%, respectively. Overall, the average tear-SFRP1 concentrations for controls and KC were 5.14 ng/µl±4.77 and 3.90 ng/µl±3.83, respectively. The average total protein concentrations of control and KC tears were 10.05 µg/µl±4.42 and 11.20 µg/µl±4.82. No significant difference in tear SFRP1 or total tear-protein concentration was found between groups (p=0.09 and p=0.376).

### Comparison of age-matched control and KC levels of SFRP1 and total protein

As there was a significant difference in age between control and KC groups when all samples were included, we reassessed tear-SFRP1 and total tear-protein levels for age-matched samples within our cohort. We analyzed samples in the 20- to 30-year age group (control n=20; KC n=19).

**Figure 2:**
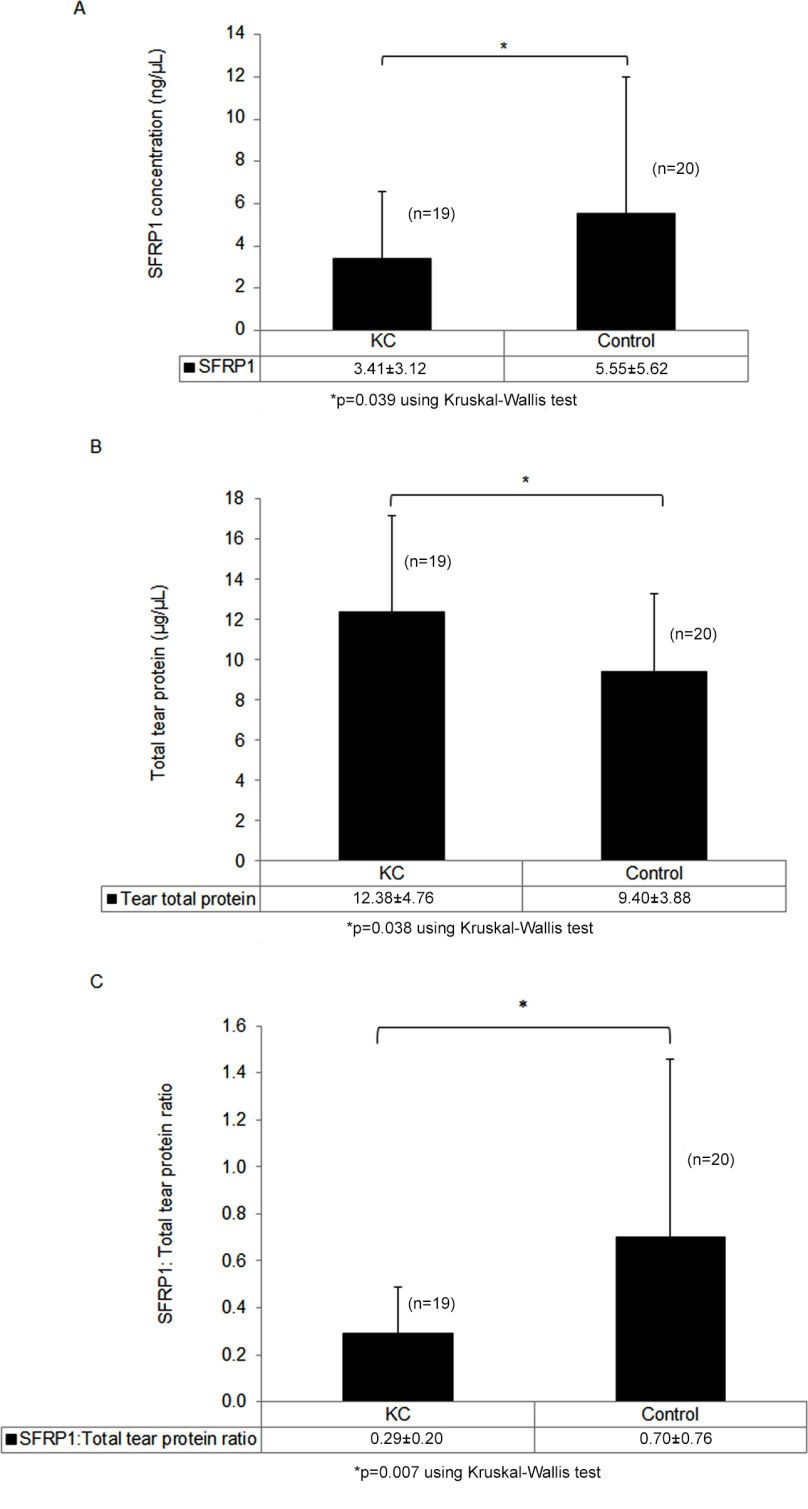
Tear secreted frizzled-related protein 1 (SFRP1) and ratio of SFRP1/total tear protein were significantly lower in age matched KC, compared to control patients. **A**: Tear SFRP1 concentration was significantly lower in age-matched KC than controls. **B**: Total tear-protein concentration in age-matched KC was significantly higher than controls. **C**: Ratio of SFRP1/ total tear protein was significantly lower in age-matched KC compared to controls. Error bar: standard deviation.

Tear SFRP1, in this age cohort, was significantly higher in controls, compared to KC (p=0.039; Figure 2A). Total tear protein was significantly lower in controls, compared to KC (p=0.038 Figure 2B). The ratio of tear SFRP1/total tear protein (ng/µg) was significantly decreased in KC, compared to controls (p=0.007, Figure 2C). No significant association between tear SFRP1 and total tear protein was detected (p=0.402), indicating that KC was the most likely factor contributing to differences in tear SFRP1 and total tear protein between the two groups.

### Tear SFRP1 and total protein versus KC Grade

**Figure 3:**
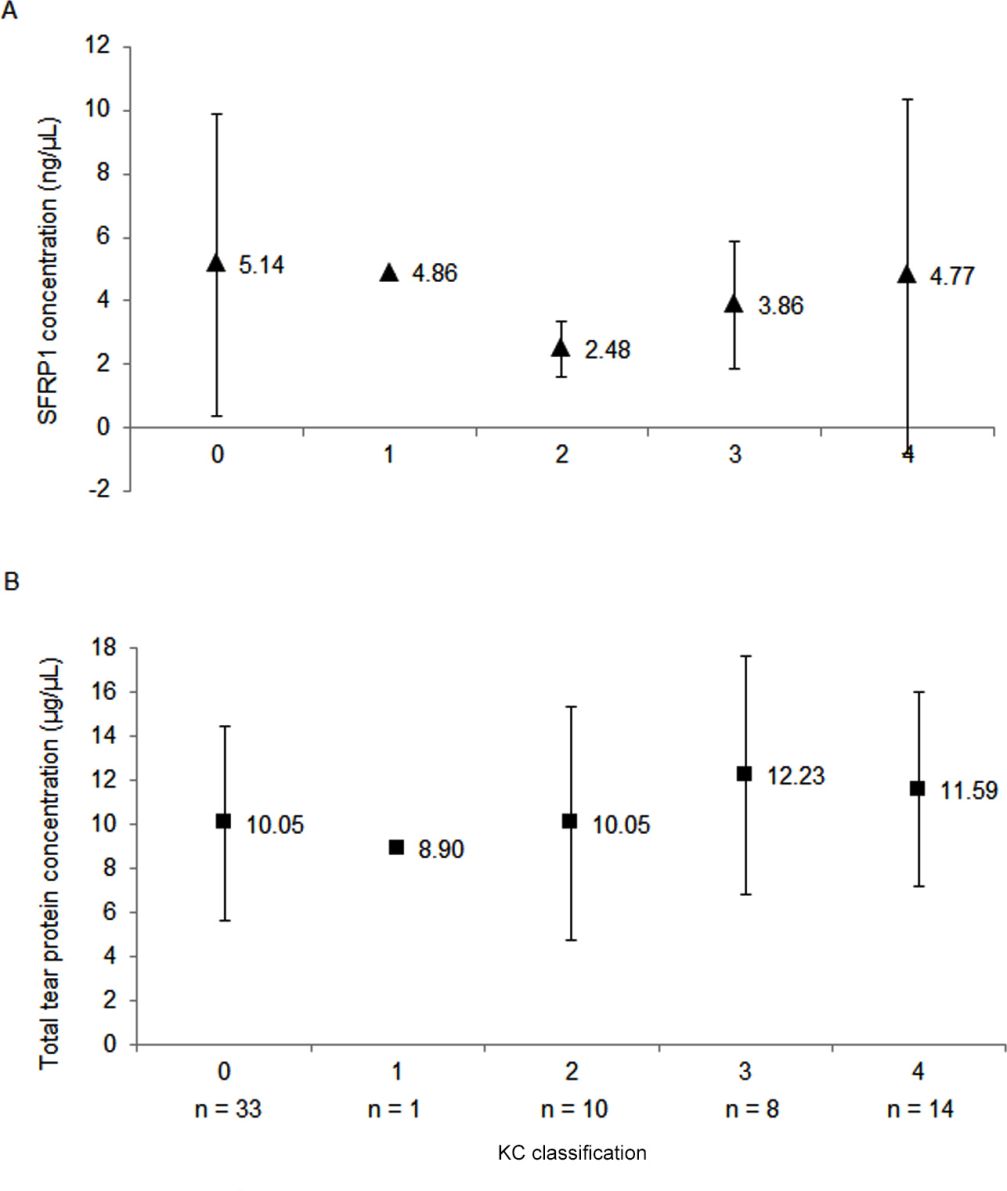
Tear secreted frizzled-related protein 1 (SFRP1) concentration was highest in controls (Grade 0), compared to individual KC grades whereas the total tear-protein concentration was highest in KC Grade 3 and 4. **A**: Tear-SFRP1 concentration for control and KC patients showing that controls had the highest concentration. **B**: Total tear-protein concentration for control and KC showing highest concentration in advanced KC (Grade 3 and 4). Error bar: standard deviation.

All subjects were included for this analysis, with the control group classified as Grade 0. As sample numbers in each KC grade (Grade 1 to 4) were small, graphical representation was used for tear-SFRP1 and total tear-protein concentrations (Figure 3). The average tear-SFRP1 concentration was highest in controls (Grade 0, 5.14 ng/µl±4.80), and reduced in individual KC grades. Within the KC grades, tear-SFRP1 levels were higher in KC Grades 1 and 4 than in KC Grades 2 and 3 (Figure 3A).

The total tear-protein concentration for KC grades differed, compared to tear SFRP1. Average total tear protein was lowest in controls (Grade 0), with KC Grades 1 and 2 lower than KC Grades 3 and 4 (Figure 3B).

## Discussion

As evidenced by the low CV%, we have successfully developed a sensitive and reliable ELISA to measure human tear SFRP1, and established that SFRP1 is normally present in tears at a nanogram-per-microliter level (a moderate abundance protein). Consistent with our preliminary immunoblotting study of tears in a smaller cohort [11], SFRP1 levels were reduced in KC tears, compared to controls, and this was significant for age-matched samples. SFRP1 levels were further reduced in tears from patients with moderate KC. This finding in tears does contrast with our earlier SFRP1 gene array and immunohistochemistry study that found significantly more SFRP1 mRNA and protein expression in KC corneal epithelium, compared to controls [4].

Unlike intracellular SFRP1 detected within the cornea, tear SFRP1 may be secreted by the corneal epithelium and other anterior eye tissues, including the lacrimal gland, accessory glands, and palpebral and bulbar conjunctival epithelium, where the SFRP1 mRNA has been detected [12]. In addition, the abundance of proteins detected biologically is not necessarily correlated to the proteins’ mRNA levels, most likely because of transcription/translation rates, post-translational modifications and protein turnover rates [13]. In fact, secreted proteins have been found to have a low mRNA and protein correlation [14].

Tear-SFRP1 protein levels could also be influenced by protein–protein interactions. Binding of secreted SFRP1 to cell-surface proteins, including Wnts, Fzds, or other proteins, may affect detectable levels of secreted proteins. Information is limited regarding SFRP, Wnt, and Fzd expression in normal human corneas and in KC, and the interactions and functions of Wnts and SFRPs are complex [5]. A recent study presented evidence for Wnt pathway-associated gene and protein expression in the adult human anterior eye, showing different patterns of Wnt, SFRP3, and SFRP5 gene expression in the limbal region, versus in the central cornea [15].

Studies using various cancer cells have consistently suggested that SFRP1 can function in both a paracrine [16] and autocrine [17] fashion. As such, SFRP1 production and release via cellular secretory pathways may also affect detection in tears. A recent in vitro study found that fibroblasts and epithelial cells, induced to undergo cellular senescence following DNA damage, showed increased intracellular SFRP1 when treated with brefeldin A, an inhibitor of protein transport from the endoplasmic reticulum to the Golgi [18]. However, this accumulation was not observed within senescent cells in the absence of brefeldin A, suggesting a rapid secretion of SFRP1 [18]. In vitro studies have also found that secreted SFRP1 can bind to the extracellular surface, including extracellular matrix proteins, and that heparin can induce increased extracellular levels of free SFRP1 [18,19]. Interestingly, the concentration of recombinant SFRP1 used to induce in vitro cellular senescence was approximately 0.5 ng/µl [18], suggesting that the levels of SFRP1 detected in tears (approximately 5 ng/µl in our study) may be functionally significant. The underlying processes that lead to differences in secreted tear SFRP1 versus intracellular corneal epithelium SFRP1, and whether levels of tear SFRP1 in KC are functionally significant, remain to be explored.

Apoptosis has also been observed in KC, mostly in anterior stromal keratocytes [20] and corneal epithelium [10], and increased corneal epithelium expression of SFRP1 may be related to apoptosis [5]. Alternatively, SFRP1 may also be antiapoptotic and have a protective mechanism, as reported by Zhou and Beuerman [21], who suggested that SFRP1 might protect corneal epithelial cells from benzalkonium chloride-induced apoptosis.

Several scenarios may be considered related to the role(s) of SFRP1 in KC. For example, accumulation of SFRP1 in compromised corneal epithelial cells secondary to impaired secretory pathways may induce epithelial apoptosis; this may also be sufficient to reduce tear SFRP1, compared to controls. Normal tear SFRP1 could also contribute to human corneal homeostasis, including maintaining limbal epithelial integrity, as suggested recently for other Wnt signaling molecules [15]. The potential role(s) of SFRPs in normal corneas and in the pathogenesis of KC are clearly complex, and require further research.

We also detected significantly higher levels of total tear protein in KC, compared to controls. Previous reports of total tear protein in KC compared to that in controls are equivocal. Pannebaker et al. [22] reported total tear-protein levels for KC and control patients similar to those in the current study. However, Balasubramanian et al. [23,24] and Acera et al. [25] detected significantly lower total protein levels in KC, compared to control tears. Various factors can affect total tear-protein concentration, including collection method (capillary tube versus Schirmer’s strip), type of tears collected (reflex versus basal), time of day and duration of tear collection, patient age, contact lens wear, storage methods, and the method used to assay total protein [21]. Our study and the published studies [22-25] all used the capillary-tube method to collect tears, which is thought the best way to avoid cellular protein contamination of the samples. Tear samples were stored at −80 °C, following collection, in all studies. The protein assay method used was different; Pannebaker et al. [22] and Acera et al. [25] used a Bradford protein assay, Balasubramanian et al. [23,24] used a BCA assay, and we used a FluoroProfile assay. These methods can generate different absolute tear-protein concentrations [26]; however ,the results should be correlated [26,27]. One possible factor contributing to differences between these studies is patient age. Our age-matched patients were 20 to 30 years of age, while in the other studies, patients were >30 years of age. The time taken for tear collection may also influence total tear protein. In their study, Balasubramanian et al. [23,24] reported collecting tears at a flow rate of <1 µl/min. We collected tears in <15 min to reduce surface dryness. Pannebaker et al. [22] reported total tear-protein concentration/total volume collected. To minimize the potential variations in tear characteristics that were not KC-related, such as reflex tearing or ocular surface dryness, we also compared tear SFRP1 to total tear protein for KC and controls, similar to Lee et al. [28]. Lee et al., [28] also used the ratio of their protein of interest to total tear protein method, as this can minimize the variations caused by possible reflex tearing during collection. This was significantly reduced in KC, compared to age-matched controls. The significance of total tear protein to the pathogenesis of KC remains to be established; however ,these observations highlight the importance of using standardized tear-collection protocols and protein assays when comparing between studies.

In conclusion, we have successfully developed an ELISA to measure SFRP1, and have established that this is a moderate-abundance protein in tears. Tear SFRP1 levels alone do not provide an obvious biomarker for KC; however, our results do provide further evidence that tear-protein profiles are altered in KC.
